# Pre-surgery statin use and outcomes in operated chronic subdural haematomas: a nationwide cohort study

**DOI:** 10.1093/ageing/afag296

**Published:** 2026-10-22

**Authors:** Jussi P Posti, Aleksi Tornio, Jori O Ruuskanen, Rahul Raj, Teemu Luoto, Pihla Tommiska, Ville Kytö

**Affiliations:** Neurocenter, Department of Neurosurgery, Turku University Hospital, Turku, Finland; Neurosurgery, Clinical Neurosciences, University of Turku, Turku, Finland; Institute of Biomedicine, University of Turku, Turku, Finland; Unit of Clinical Pharmacology, Turku University Hospital, Turku, Finland; Neurocenter, Department of Neurology, Turku University Hospital, Turku, Finland; Neurology, Clinical Neurosciences, University of Turku, Turku, Finland; Department of Neurosurgery, Helsinki University Hospital and University of Helsinki, Helsinki, Finland; Department of Neurosurgery, Tampere University Hospital, Tampere University, Tampere, Finland; Department of Neurosurgery, Helsinki University Hospital and University of Helsinki, Helsinki, Finland; Clinical Research Center, Turku University Hospital, Turku, Finland; Heart Centre, Turku University Hospital and University of Turku, Turku, Finland

**Keywords:** chronic subdural haematoma, statins, mortality, reoperation, recurrence, length of stay, registry study, older people

## Abstract

**Background:**

Chronic subdural haematoma (cSDH) is common in older adults, and medication exposures may influence postoperative outcomes. We assessed whether pre-surgery statin use is associated with short- and long-term outcomes.

**Methods:**

We conducted a nationwide registry study of adults undergoing surgical evacuation of traumatic or non-traumatic cSDH (6/2004–12/2018) in Finnish university hospitals. Pre-surgery statin use was defined from national prescription records using a pill-counting approach. The primary outcome was 30-day all-cause mortality. Secondary outcomes were 30-day reoperation and length of hospital stay. Tertiary outcomes were 1-year mortality and reoperation. Mortality was analysed using Cox regression; reoperation with Fine–Gray models accounting for competing risk of death.

**Results:**

The cohort included 9001 patients, of whom 2450 (27.2%) used statins. Thirty-day mortality was 3.3% in statin users and 4.5% in non-users, with lower adjusted mortality among statin users (HR 0.57; 95% CI 0.44–0.73). Reoperation within 30 days (*n* = 897) was similar between groups (sub-distribution HR 1.08; 95% CI 0.97–1.21). Hospital stay was shorter in statin users (22.1 ± 62.7 vs 26.3 ± 77.5 days; adjusted *P* = .002). At 1 year, mortality remained lower in statin users (13.3% vs 15.4%; HR 0.66; 95% CI 0.58–0.75), with no difference in reoperation (sub-distribution HR 1.09; 95% CI 0.96–1.22).

**Conclusion:**

In this nationwide cohort of surgically treated cSDH patients, pre-surgery statin use was associated with lower short- and long-term mortality and shorter hospital stay, while reoperation rates were similar between statin users and non-users.

## Key points

- In a nationwide cohort of 9001 surgically treated cSDH patients, 27.2% used statins pre-surgery.
- Pre-surgery statin use was associated with lower adjusted 30-day (hazard ratio 0.57) and 1-year mortality (hazard ratio 0.66).
- Hospital admission duration after surgery was shorter in statin users.
- Reoperation rates were similar in statin users and non-users at both 30 days and 1 year.
- These findings suggest an association between pre-surgery statin use and improved survival after cSDH evacuation.

## Introduction

Chronic subdural haematoma (cSDH) is one of the most common neurosurgical conditions worldwide, and its incidence continues to rise with ageing populations and increasing use of antithrombotic therapies [1–4]. Surgical evacuation remains the standard treatment for symptomatic cSDH, and postoperative recurrence occurs in approximately 5%–30% of patients, and both short- and long-term mortality remain clinically significant, particularly in older adults [2, 5, 6].

Current concepts characterise cSDH not as a static chronic blood accumulation, but as a dynamic inflammatory and angiogenic disease. It has been suggested that fragile haematoma membrane microvasculature, local inflammation and dysregulated fibrinolysis promote exudation and episodic bleeding, resulting in haematoma growth and recurrence [7–9]. This pathophysiological context has prompted interest in adjunctive medical therapies aimed at reducing haematoma growth and improving outcomes beyond surgical evacuation alone [1, 10].

Statins, particularly atorvastatin, have emerged as candidate pharmacologic agents due to pleiotropic effects such as anti-inflammation, endothelial activity and modulation of angiogenesis, which are potentially relevant mechanisms in cSDH development [8, 11]. Several studies have examined atorvastatin as conservative therapy or as an adjunct to surgery in the treatment of cSDH. Some studies have reported reduced recurrence rates and favourable radiological or functional outcomes [12–15]. A randomised clinical trial demonstrated that short-term atorvastatin therapy was feasible and associated with haematoma resolution in selected patients [14], although subsequent systematic reviews and meta-analyses have reported mixed conclusions, reflecting heterogeneity in study populations, endpoints and treatment timing [9, 10, 16].

Most previous studies have focused on initiating statin therapy after cSDH diagnosis, often with emphasis on recurrence or radiological endpoints [12–15]. In contrast, the potential impact of pre-surgery statin use—that is, the effect of statins on condition development and on possibly modulating recovery—on clinically meaningful outcomes after surgical treatment of cSDH has received limited attention. Prospective and retrospective studies in traumatic brain injury (TBI) have suggested associations between pre-surgery statin use and improved survival, supporting the need to examine whether similar associations exist in surgically treated cSDH [17–21].

Therefore, we conducted a nationwide cohort study to evaluate the association between pre-surgery statin use and outcomes after surgical evacuation of cSDH. The primary outcome was 30-day all-cause mortality. Secondary outcomes were 30-day reoperation for cSDH and length of hospital stay. Tertiary outcomes were 1-year all-cause mortality and 1-year reoperation. Using comprehensive national registry data, we aimed to clarify whether pre-existing statin therapy is associated with improved postoperative outcomes in this common and increasingly prevalent neurosurgical condition. We hypothesised that pre-surgery statin use would be associated with improved postoperative outcomes, particularly regarding mortality, while effects on reoperation rates were expected to be less pronounced.

## Materials and methods

### Patients

Patients aged 18 years or older with traumatic or non-traumatic cSDH who underwent surgical evacuation between 1 July 2004, and 31 December 2018 were retrospectively identified from the nationwide Care Register for Health Care (CRHC). Only patients treated at university hospitals (*n* = 5) were included, as all intracranial neurosurgical procedures in Finland are performed at these hospitals. Eligible cases were defined by the presence of an ICD-10 (International Statistical Classification of Diseases and Related Health Problems, Tenth Revision) code S06.5 or I62.0 recorded as any diagnosis, in combination with NOMESCO (Nordic Medico-Statistical Committee) procedure codes AAD10 and/or AAD12 [2]. For each patient, the index operation was defined as the first recorded cSDH operation during the study period, and only this operation was included in the analyses. Previous cSDH operations before the start of the study period could not be identified. Hospital transfers, including those from university hospitals to central and regional hospitals and health centre wards related to a particular hospitalisation episode, were combined as one admission. cSDH is usually recorded under S06.5 when a traumatic cause is documented and under I62.0 when it is not; both codes were therefore used. S06.5 belongs to the ICD-10 chapter for intracranial injury (S06.^*^), whose validity in the CRHC has previously been assessed by medical record review, with a positive predictive value of 0.98 [22].

### Outcomes

The primary outcome was all-cause mortality at 30 days after haematoma evacuation. Secondary outcomes were reoperation for cSDH at 30 days after haematoma evacuation and the duration of hospital admission after the index operation, including ward stays and interhospital transfers. Tertiary outcomes were all-cause mortality and reoperation for cSDH at 1 year after the index procedure. Mortality follow-up was available until 31 December 2019, and reoperation follow-up until 31 December 2018.

### Medications

Pre-surgery statin use was determined from pharmacy purchases within 90 days before cSDH admission using Anatomical Therapeutic Chemical (ATC) codes (Supplementary Appendix 1). To estimate statin use on the day of admission, we used a daily pill-counting method, assuming a dose of one pill per day. Statin therapy was classified by solubility (lipophilic or hydrophilic) and intensity (high or non-high) (Supplementary Appendix 1) [23]. The statin used was defined as the most recent statin purchased before cSDH admission. In Finland, statins are available only by prescription, and all pharmacy purchases are recorded in a national database used in this study [24]. Patients with missing follow-up data (*n* = 19) and those with uncertain statin use—defined as patients who purchased a statin within 90 days before admission, but whose purchased supply did not cover the day of neurosurgical admission (*n* = 49)—were excluded.

### Comorbidities

Baseline comorbidities were identified from linked nationwide healthcare registries using ICD-10 diagnosis codes recorded at or before the index admission. Comorbidity definitions followed established Finnish registry-based methodology and included cardiovascular, cerebrovascular, metabolic, pulmonary, renal, hepatic, malignant, psychiatric and substance abuse-related conditions, as previously described [25].

### Standard protocol approvals, registrations and patient consents

Information on hospital admissions, diagnoses and procedures was obtained from the CRHC. Data on prescription drug purchases—including ATC codes, strength, quantity and date—as well as reimbursement permissions and information from the Finnish Cancer Registry, were provided by the National Institute for Health and Welfare of Finland/Findata (permission No. THL/164/14.02.00/2021). Mortality data were obtained from Statistics Finland (permission No. TK-53-484-20). As the collection and reporting of these nationwide registries are mandated by law, population coverage is nearly complete and follow-up was available for all included patients. As this was a retrospective nationwide registry study, ethics committee approval and individual informed consent were not required, and participants were not contacted. Individual-level data used in the study are accessible with permission from Findata (findata.fi). Processing of personal data was based on the legal grounds of public interest and scientific research in accordance with the EU General Data Protection Regulation (2016/679, Article 6(1)(e) and Article 9(2)(j)) and the Finnish Data Protection Act (Sections 4 and 6).

### Statistical analysis

Baseline characteristics were compared using Chi-square tests and *t* tests, as appropriate. Mortality was analysed using Kaplan–Meier estimators and Cox proportional hazards regression. Reoperation was assessed using cumulative incidence functions and Fine–Gray regression models, accounting for the competing risk of death. Length of hospital admission was analysed using linear regression with a log-transformed and standardised dependent variable.

All regression analyses were adjusted for baseline age, sex, alcohol abuse, atrial fibrillation or flutter, cerebrovascular disease, chronic pulmonary disease, coagulopathy, coronary artery disease, dementia, diabetes, heart failure, heart valve disease, hypertension, liver disease, malignancy, paralysis, peripheral arterial disease, psychotic disorder, rheumatic disease, renal failure, year of cSDH operation (categorised as 2004–2008, 2009–2013 or 2014–2018) and treating hospital. Covariates were selected a priori based on previous knowledge and clinical judgement. Data on frailty and baseline functional status are not recorded in the Finnish national registries. The covariate set includes determinants of functional dependency (dementia, paralysis, malignancy, heart failure, renal failure, chronic pulmonary disease and alcohol abuse disorder) as partial proxies. Proportional hazards assumptions were evaluated using Schoenfeld residuals. The *E*-value, representing the extent to which unmeasured confounding would have to be associated with both the exposure (statin use) and the outcome to nullify the observed exposure–outcome association, was calculated [26].

Results are presented as means, percentages, hazard ratios (HRs) or sub-distribution hazard ratios (sHRs) with 95% confidence intervals (CIs) or ± SD, as appropriate. Statistical significance was defined as a *P* value <.05. All analyses were performed using SAS version 9.4 (SAS Institute Inc., Cary, NC, USA).

## Results

The total study population included 9001 patients operated on for cSDH, of whom 2450 (27.2%) were classified as pre-surgery statin users. The mean age was 73.3 years (SD 12.6), and 68.1% were male. Statin users were older and had a higher overall comorbidity burden than non-statin users (Table 1). Patients with alcohol abuse disorder were less likely to use statins. The proportion of patients using statins increased during the study period. Simvastatin was the most used statin (Supplementary Appendix 2). High-intensity statin therapy was used in 9.4% of statin users. The statin used was lipophilic in 89.2% of cases and hydrophilic in 10.8%.

**Table 1:** Baseline features of patients with operated cSDH with and without pre-surgery statin use.

Variable	All patients *n* = 9,001	Statin *n* = 2,450	No Statin *n* = 6,551	*P* value
Age, years (SD)	73.3 (12.6)	75.9 (9.1)	72.3 (13.5)	<0.0001
Male	68.1 %	69.3 %	67.6 %	0.152
Comorbidities				
Alcohol abuse	9.6 %	4.6 %	11.5 %	<0.0001
Arterial disease	56.5 %	76.2 %	49.3 %	<0.0001
Cerebrovascular	42.6 %	51.7 %	39.0 %	<0.0001
Coronary artery	25.8 %	48.4 %	17.4 %	<0.0001
Peripheral	5.8 %	10.1 %	4.1 %	<0.0001
Atrial fibrillation	23.7 %	32.0 %	20.6 %	<0.0001
Chronic pulmonary disease	11.4 %	12.9 %	10.8 %	0.007
Coagulopathy	1.1 %	1.1 %	1.1 %	0.909
Dementia	12.2 %	13.7 %	11.7 %	0.008
Diabetes	18.2 %	31.4 %	13.3 %	<0.0001
Heart failure	13.4 %	19.0 %	11.3 %	<0.0001
Heart valve disease	6.6 %	10.2 %	5.2 %	<0.0001
Hypertension	45.7 %	61.3 %	39.9 %	<0.0001
Liver disease	2.3 %	1.6 %	2.6 %	0.004
Malignancy	18.0 %	19.6 %	17.4 %	0.013
Paralysis	1.1 %	1.1 %	1.1 %	0.714
Psychotic disorder	4.4 %	3.8 %	4.6 %	0.068
Rheumatic disease	4.7 %	5.2 %	4.5 %	0.195
Renal failure	2.6 %	4.0 %	2.0 %	<0.0001
Year of operation^*^				<0.0001
2004–2008	24.5 %	19.8 %	80.2 %	
2009–2013	34.2 %	28.4 %	71.6 %	
2014–2018	41.3 %	30.7 %	69.3 %	

Values are percentages within each column unless otherwise indicated; ^*^In the *Year of operation* section, the *All patients* column gives the proportion of the whole cohort operated on during each period, whereas the *Statin* and *No statin* columns give the distribution of statin use within each period.

**Figure 1:**
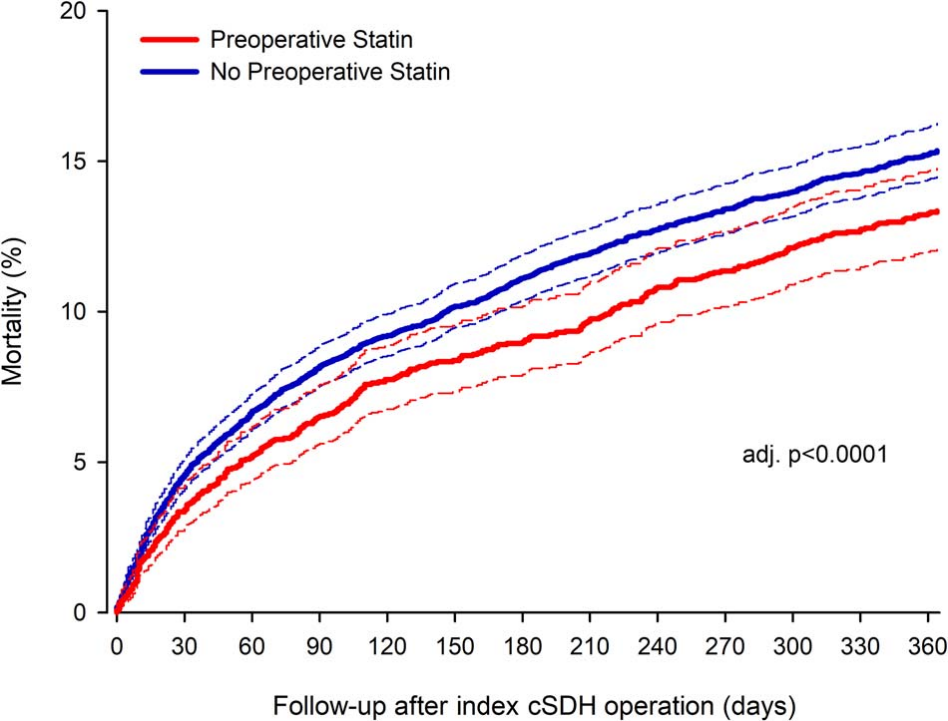
Mortality after haematoma evacuation for chronic subdural haematoma in patients with pre-surgery statin use and patients without statin. Dashed lines represent 95% CIs.

### Thirty-day mortality and reoperation

A total of 377 patients died within 30 days of the index procedure, corresponding to a mortality rate of 4.2% (Fig. 1). The 30-day mortality rate was 3.3% among statin users and 4.5% among non-statin users (unadjusted *P* = .015). In multivariable-adjusted analyses, pre-surgery statin use was associated with lower 30-day mortality compared with no statin use (adjusted HR 0.57; 95% CI 0.44–0.73; *P* < .0001). The association was similar across statin subgroups defined by solubility and intensity (Table 2). The *E*-value for the observed association with 30-day mortality was 2.90 (95% CI 2.08–3.97).

Reoperation for cSDH was performed in 897 patients within 30 days of the index procedure (Fig. 2). The cumulative incidence of reoperation at 30 days was 10.5% in statin users and 9.8% in non-statin users (unadjusted *P* = .332). In multivariable-adjusted competing risk analyses accounting for death, statin use was not associated with reoperation at 30 days (adjusted sHR 1.08; 95% CI 0.97–1.21; *P* = .169). Results were consistent in subgroup analyses by statin solubility and intensity (Supplementary Appendix 3).

**Figure 2:**
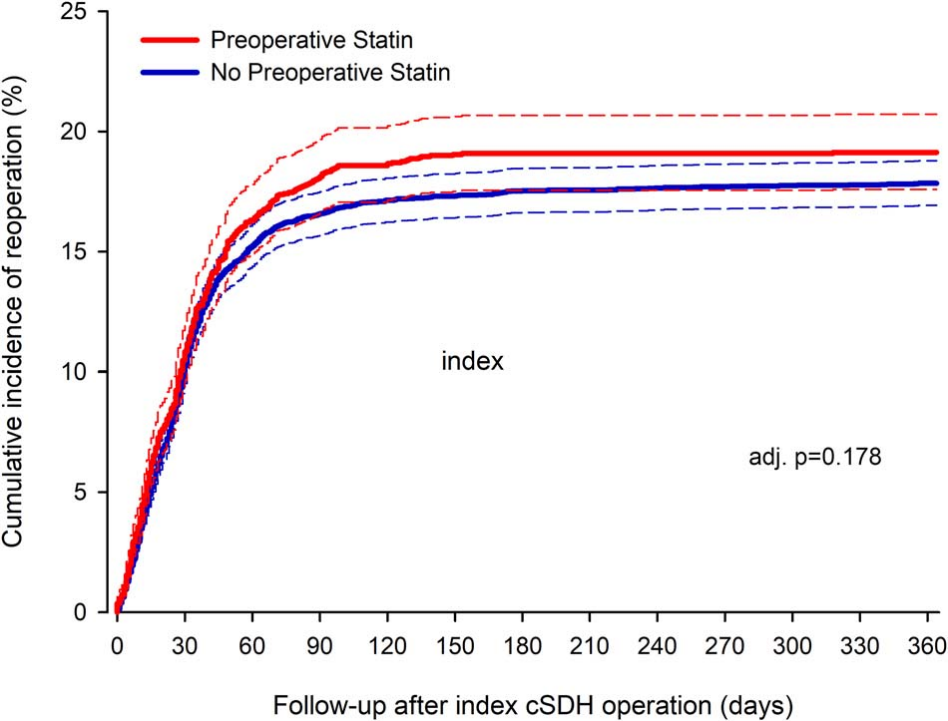
Cumulative incidence of reoperation after index haematoma evacuation for chronic subdural haematoma in patients with pre-surgery statin use and patients without statin. Dashed lines represent 95% CIs.

### Length of hospital admission

The mean length of combined hospital admission after the index operation was 25.2 ± 73.8 days (range 1–1997 days). Hospital stay was shorter among statin users (22.1 ± 62.7 days) than among non-statin users (26.3 ± 77.5 days; unadjusted *P* = .012). In multivariable-adjusted analyses, pre-surgery statin use was associated with a shorter duration of hospital admission after surgery (adjusted *P* = .002). Hospital stay was shorter among users of hydrophilic statins (adjusted *P* = .006), lipophilic statins (adjusted *P* = .009) and non–high-intensity statins (adjusted *P* = .002) compared with no statin use, whereas no difference was observed for high-intensity statin use versus no statin use (adjusted *P* = .422).

**Table 2:** Association of pre-surgery statin use with 30-day and 1-year mortalities by statin solubility and treatment intensity in multivariable-adjusted analyses.

	Statin vs. no statin			
	30-day mortality		1-year mortality	
	HR (95 % CI)	*P* value	HR (95 % CI)	*P* value
All patients	0.57 (0.44–0.73)	<0.0001	0.66 (0.58–0.75)	<0.0001
Statin solubility				
Lipophilic	0.54 (0.41–0.71)	<0.0001	0.66 (0.57–0.76)	<0.0001
Hydrophilic	0.82 (0.46–1.48)	0.517	0.68 (0.49–0.96)	0.030
Statin treatment intensity				
High	0.40 (0.17–0.97)	0.042	0.54 (0.36–0.82)	0.003
Non–high	0.58 (0.44–0.75)	<0.0001	0.67 (0.58–0.77)	<0.0001

### One-year mortality and reoperation

A total of 1333 patients died within 1 year of the index procedure, corresponding to a mortality rate of 14.8% (Fig. 1). The 1-year mortality rate was 13.3% among statin users and 15.4% among non-statin users (unadjusted *P* = .014). In multivariable-adjusted analyses, pre-surgery statin use was associated with lower 1-year mortality (adjusted HR 0.66; 95% CI 0.58–0.75; *P* < .0001). The *E*-value for the observed association with 1-year mortality was 2.40 (95% CI 2.00–2.84). Associations were consistent across statin subgroups defined by solubility and intensity (Table 2).

Reoperation for cSDH was performed in 1629 patients within 1 year of the index procedure (Fig. 2). The cumulative incidence of reoperation at 1 year was 19.1% in statin users and 17.8% in non-statin users (unadjusted *P* = .151). In multivariable-adjusted competing risk analyses, pre-surgery statin use was not associated with reoperation at 1 year (adjusted sHR 1.09; 95% CI 0.96–1.22; *P* = .178). Subgroup analyses by statin solubility and intensity yielded similar results (Supplementary Appendix 3).

## Discussion

In this nationwide cohort of 9001 patients who underwent surgical evacuation of cSDH (index operation during the study period), pre-surgery statin use was associated with lower 30-day all-cause mortality, while 30-day reoperation rates were similar between statin users and non-users. Statin users also had a shorter length of hospital stay after the index operation. At 1 year, pre-surgery statin use remained associated with lower mortality, whereas reoperation rates again did not differ between groups.

The combination of lower mortality and shorter admissions, without evidence of reduced reoperation among statin users, suggests that the associations between pre-surgery statin use and improved postoperative outcomes may be mediated largely through systemic pathways or may reflect differences in overall health care and comorbidity management, rather than a direct reduction in surgically relevant recurrence requiring reoperation.

Mechanistically, cSDH is increasingly conceptualised as a chronic inflammatory and angiogenic disorder driven by haematoma membrane formation, fragile microvasculature and dysregulated inflammatory signalling [15, 27]. Evidence on these processes has motivated interest in pharmacological adjuncts, including statins and especially atorvastatin, because of their pleiotropic anti-inflammatory and endothelial-targeted effects [13, 15, 27, 28]. However, the clinical evidence base has been heterogeneous and setting-specific, and the strongest signals for benefit have often been reported in conservative treatment pathways or imaging-derived endpoints, rather than in hard postoperative endpoints [10, 13, 14, 28].

Several clinical studies, including cohorts receiving conservative and/or surgical treatment, have suggested that atorvastatin may be associated with improved resolution and reduced recurrence [8, 9, 29]. A later meta-analysis reported indications of benefit for atorvastatin across studies, although the included evidence varied in design, outcome definitions and treatment context [7]. Subsequent systematic reviews and meta-analyses have highlighted both the promise and the uncertainty: pooled estimates have suggested potential reductions in recurrence in some contexts, but subgroup findings have varied, and results may be diluted by the higher proportion of patients on antithrombotic medications in the statin groups [1, 10].

In our cohort, which was restricted to surgically treated patients, pre-surgery statin use was not associated with a reduced rate of reoperation at 30 days or 1 year. This is consistent with evidence syntheses suggesting that any effect of statins or atorvastatin on recurrence may be more apparent in conservatively managed cohorts or when using radiological endpoints, whereas the postoperative reoperation endpoint is less sensitive to modest biological effects and is more influenced by surgical, anatomical and clinical decision factors [1, 10]. The finding that lipid-related factors may still be associated with the postoperative course is supported by a study on cholesterol levels in postoperative cSDH, which reported that higher high-density lipoprotein cholesterol was associated with improved postoperative outcomes, while exposure to cholesterol-lowering medication itself did not clearly result in lower recurrence or reoperation [30].

Conservative management is often attempted in patients with minimally symptomatic cSDH; however, a substantial proportion ultimately require surgical intervention, highlighting the limited durability of non-operative strategies [16]. In this context, pharmacological adjuncts such as statins have attracted interest, not primarily as agents to prevent reoperation, but as modifiers of the biological processes underlying haematoma resolution and recovery. Recent work mapping the pharmacological landscape of cSDH similarly demonstrates that conclusions are sensitive to endpoint selection and study design, and that ranking therapies does not always translate into consistent benefit across interventional pathways [13].

The association between pre-surgery statin use and reduced mortality at both 30 days and 1 year is clinically significant but requires careful interpretation. Statin exposure is closely associated with cardiovascular disease prevention and engagement with the health system; residual confounding, including baseline function, frailty, lifestyle factors and healthy adherer effects, may persist despite extensive adjustment. People who take preventive medication are more often non-smokers and in regular contact with primary care, so they may present earlier and in better condition. Ongoing medication use may itself indicate better general health. Statin users in our cohort were nonetheless older and had more comorbidities. Frailty and baseline functional status are not recorded in the Finnish registry data, yet they are among the strongest determinants of mortality after cSDH surgery [5], and the included comorbidity covariates are imperfect proxies. Radiological features of cSDHs are also unavailable, yet they influence both postoperative outcomes and the threshold for operating. Previous registry-based evidence in acute TBI has also reported associations between pre-surgery statin exposure and improved survival, providing broader context that survival signals may arise in acute neurological injury populations even when direct lesion-related endpoints are unaffected [17].

Lower mortality without fewer reoperations does not weaken the main outcome observation. Reoperation and death are largely separate outcomes after cSDH surgery. In the FINISH trial, among patients assigned to the arm of cSDH operation without subdural irrigation, the 6-month reoperation rate was 6.0 percentage points higher, yet mortality did not differ [6]. In a post hoc study of the same trial, the leading causes of death were circulatory disease (34%), accidents (16%) and dementia (15%). Mortality rose from 4.7% in patients with no comorbidity to 46.2% in those with four, suggesting that mortality after cSDH surgery relates more to comorbidities than to the haematoma itself [31]. The current results follow this pattern: mortality was lower at both 30 days and 1 year, while reoperation was unchanged at both time points.

Antithrombotic therapy warrants separate remark as antiplatelet agents and anticoagulants were not included in this study. Statin users had higher rates of atrial fibrillation, coronary artery disease and cerebrovascular disease and would receive these medications more often, so adjustment for these indications captures much, though not all, of the associated confounding. Although antithrombotic use is an established risk factor for the development of cSDH [4], its association with postoperative recurrence is less clear, and a post hoc analysis of the FINISH trial found similar recurrence rates with and without preoperative anticoagulation for atrial fibrillation [32]. As anticoagulated patients in that analysis had more postoperative complications and worse outcomes, any unmeasured antithrombotic use among statin users would, if anything, attenuate rather than create the survival difference we observed. As antithrombotic use was not measured, however, and its prescribing and management are complex, residual confounding from this source cannot be excluded.

The strengths of this study include its nationwide design, covering all Finnish university hospitals performing intracranial neurosurgery, comprehensive linkage to prescription purchase records, the national care registry and near-complete mortality follow-up. The competing-risk approach for reoperation appropriately accounts for death, which is essential in older surgical populations.

Limitations are inherent in observational registry research. Medication exposure is inferred from purchases rather than actual use, and perioperative continuation or discontinuation cannot be directly assessed. The study examined pre-surgery statin use, and we collected data only before hospitalisation; therefore, medications administered in hospital were not included in the analyses. Because we had no access to dosing instructions, the statin dose was assumed to be one pill per day, which is the case in 95% of statin prescriptions in Finland [33]. Such misclassification is unlikely to be associated with patient outcomes, and any resulting error would tend to dilute rather than exaggerate the associations we report. Because one tablet nonetheless corresponds to different doses across statin types and intensities, exposure may be classified slightly less accurately for high-intensity regimens, and the subgroup estimates by solubility and intensity should be interpreted with corresponding caution. As discussed above, clinical and radiological detail is limited, and postoperative functional outcomes are unavailable. The reoperation endpoint captures clinically decisive treatment failure, but not radiological recurrence managed conservatively, so differences from imaging-based recurrence literature are expected. Finally, practice patterns and adjuvant strategies evolved during the long study period, although adjustment for treating hospital and operation era partially addresses secular trends. Last, an unmeasured confounder would need to be associated with both statin use and 30-day mortality with a risk ratio of at least 2.9 to fully explain this association [26]. Given the factors we could not measure (frailty, functional status, haematoma characteristics, healthcare engagement and antithrombotic use), a confounder of this magnitude cannot be considered implausible.

## Conclusion

In this nationwide cohort of surgically treated cSDH patients, pre-surgery statin use was associated with lower short- and long-term mortality and a shorter hospital stay. In contrast, statin use was not associated with a reduced need for reoperation after surgical evacuation. Overall, these findings indicate that statin exposure—whether through systemic biological effects or as a marker of overall health status and care patterns—may be linked to survival and recovery trajectories in cSDH rather than lesion-specific surgical outcomes. Residual confounding by frailty and baseline functional status cannot be excluded. Future studies should explicitly separate lesion-related endpoints from systemic postoperative outcomes, incorporate functional measures where possible and apply trial or target-trial emulation approaches tailored to surgically treated cSDH populations.

## Supplementary Material

Supplementary_materials_afag296

## Data Availability

By law, we are not permitted to disclose data to third parties. Requests to access the data set may be sent to Findata, Helsinki, Finland.
