# Supplementary material for: Pre-surgery statin use and outcomes in operated chronic subdural haematomas: a nationwide cohort study
Source: Age Ageing. 2026 Oct 22;55(10):afag296. doi: 10.1093/ageing/afag296 (PMC13688349; doi:10.1093/ageing/afag296)
Supplement: Supplementary file 1 [file supplementary_materials_afag296.docx]

**Pre-surgery Statin Use and Outcomes in Operated Chronic Subdural Haematomas: A Nationwide Cohort Study – Appendices**

Jussi P. Posti, MD, PhD¹˒²; Aleksi Tornio, MD, PhD³˒⁴; Jori O. Ruuskanen, MD, PhD⁵˒⁶; Rahul Raj, MD, PhD⁷; Teemu Luoto, MD, PhD⁸; Pihla Tommiska, MD, PhD⁷; Ville Kytö, MD, PhD⁹˒¹⁰

**Affiliations**

¹Neurocenter, Department of Neurosurgery, Turku University Hospital, Turku, Finland; ²Neurosurgery, Clinical Neurosciences, University of Turku, Turku, Finland; ³Institute of Biomedicine, University of Turku, Turku, Finland; ⁴Unit of Clinical Pharmacology, Turku University Hospital, Turku, Finland; ⁵Neurocenter, Department of Neurology, Turku University Hospital, Turku, Finland; ⁶Neurology, Clinical Neurosciences, University of Turku, Turku, Finland; ⁷Department of Neurosurgery, Helsinki University Hospital and University of Helsinki, Helsinki, Finland; ⁸Department of Neurosurgery, Tampere University Hospital, Tampere University, Tampere, Finland; ⁹Clinical Research Center, Turku University Hospital, Turku, Finland; ¹⁰Heart Centre, Turku University Hospital and University of Turku, Turku, Finland

**Corresponding author’s name and current affiliation**

Jussi Posti, Neurocenter, Department of Neurosurgery, Turku University Hospital, Hämeentie 11, FI-20521, Turku, Finland; jussi.posti@utu.fi

|  | **Intensity** | |  |  |
| --- | --- | --- | --- | --- |
|  | **High** | **Non-High** | **Solubility** | **ATC codes** |
| Atorvastatin | 40–80 mg | 10–20 mg | Lipophilic | C10AA05, C10BA05, C10BX03, C10BX08, C10BX11, C10BX12, C10BX15 |
| Fluvastatin | - | 20–80 mg | Lipophilic | C10AA04 |
| Lovastatin | - | 20–40 mg | Lipophilic | C10AA02, C10BA01 |
| Pravastatin | - | 10–40 mg | Hydrophilic | C10AA03, C10BA03, C10BX02 |
| Rosuvastatin | 20–40 mg | 10 mg | Hydrophilic | C10AA07, C10BA06, C10BX05, C10BX07, C10BX09, C10BX10, C10BX13, C10BX14 |
| Simvastatin | 80 mg | 20–60 mg | Lipophilic | C10AA01, C10BA02, C10BA04, C10BX01, C10BX04 |

**Appendix 1.** Classification of statin medications used by patients with chronic subdural hematoma. Therapy intensity, solubility, and Anatomical Therapeutical Classification (ATC) codes used for drug detection. Cerivastatin (ATC-code C10AA06) or pitavastatin (C10AA08) were not used by the study patients.

|  |  | **Intensity** | |
| --- | --- | --- | --- |
|  | **Total** | **High** | **Non-High** |
| Simvastatin | 58.8 % | 0.6 % | 99.4 % |
| Atorvastatin | 24.9 % | 26.7 % | 73.3 % |
| Rosuvastatin | 7.7 % | 30.7 % | 69.3 % |
| Fluvastatin | 3.7 % | - | 100 % |
| Pravastatin | 3.1 % | - | 100 % |
| Lovastatin | 1.8 % | - | 100 % |

**Appendix 2.** Distribution of statins used at the time of chronic subdural hematoma operation in the original cohort.

|  | | **Statin vs. no statin** | | | |
| --- | --- | --- | --- | --- | --- |
|  | | **30-day reoperation** | | **1-year reoperation** | |
|  | | **sHR (95 % CI)** | ***P* value** | **sHR (95 % CI)** | ***P* value** |
| All patients | | 1.08 (0.97–1.21) | 0.169 | 1.09 (0.96–1.22) | 0.178 |
| Statin solubility | |  |  |  |  |
|  | Lipophilic | 1.08 (0.92–1.27) | 0.337 | 1.09 (0.96–1.23) | 0.177 |
|  | Hydrophilic | 0.97 (0.64–1.46) | 0.869 | 1.06 (0.79–1.43) | 0.686 |
| Statin treatment intensity | |  |  |  |  |
|  | High | 1.34 (0.91–1.98) | 0.145 | 1.32 (0.98–1.76) | 0.106 |
|  | Non-high | 1.05 (0.89–1.23) | 0.571 | 1.06 (0.94–1.20) | 0.354 |

**Appendix 3.** Association of statin use at the time of operation of chronic subdural hematoma with 30-day and one-year reoperation by statin solubility and treatment intensity in multivariable adjusted competing risk analyses.
